# Health care utilization, somatic and mental health distress, and well-being among widowed and non-widowed female survivors of war

**DOI:** 10.1186/1471-244X-12-39

**Published:** 2012-05-11

**Authors:** Nexhmedin Morina, Paul M G Emmelkamp

**Affiliations:** 1 Department of Clinical Psychology, University of Amsterdam, Weesperplein 4, 1018, XA, Amsterdam, The Netherlands

## Abstract

**Background:**

The aim of the study was to assess levels of somatic and mental health distress, well-being, AS WELL AS utilization of primary and specialist health care services among war-related widowed and non-widowed female civilian survivors of war.

**Methods:**

100 war-related widowed lone mothers and 106 non-widowed mothers who had experienced the Kosovo war ten years previously participated in the study. Measures of somatic, depressive, post-traumatic stress, anxiety, and grief complaints, subjective well-being, and utilization of health care services during the previous three months were used.

**Results:**

Compared to non-widowed mothers, widowed lone mothers reported significantly higher levels of somatic, depressive, post-traumatic stress, and anxiety complaints. Further, they reported significantly lower levels of subjective well-being as composed of positive and negative affect and satisfaction with life. More than half of both widowed and non-widowed mothers reported utilization of health care services during the last three months, without significant differences between the groups. However, only three percent of widowed lone mothers and four percent of non-bereaved mothers reported utilization of mental health services during the last three months, despite high levels of mental health distress especially among widowed lone mothers. Among widowed lone mothers, severity of prolonged grief symptoms significantly predicted number of contacts of specialist health care use over and above sociodemographic variables, number of war-related events, and other psychopathology.

**Conclusion:**

War-related widowed lone mothers suffer from elevated somatic and mental distress even a decade after the war. The tiny proportion of widowed lone mothers in use of mental health services can be seen as a reflection of lack of previous and current mental health services to meet mental health needs of this population.

## Background

Since the First World War when opposing armies still fought along static lines of defense, the nature of warfare has drastically changed. Civilians are increasingly exposed to violations of human rights and therefore make up the absolute majority of casualties [1]. Research indicates that exposure to war-related traumatic experiences among civilian war survivors can lead to elevated rates of depression and anxiety disorders [2]. However, exposure to war-related experiences can comprise a broad variety of events and the very nature of certain war-related experiences has received little scientific attention. Based on results from bereavement research in general [3], it can be assumed that war-related bereavement constitutes a significant risk factor for distress and dysfunction above and beyond other war-related traumatic experiences. Research in Western countries has shown that bereavement is associated with higher prevalence of physical and mental symptoms and with increased use of medical services [3]. Compared to other causes of death, violent death of a loved one has been associated with poorer health [4-6].

Despite the fact that killing defines the very nature of war like no other feature and despite growing evidence on the detrimental effect of bereavement, research on the impact of loss of family members due to war-related violence on mental health among civilian war survivors has only recently started to emerge [7-10]. In order to better understand the role of war-related bereavement above and beyond exposure to other war-related events, studies comparing war survivors with and without war-related bereavement might prove useful. Recently, Morina et al. [9] reported that major depressive episode and anxiety disorders were significantly more prevalent among young adults who had lost their father due to war-related violence in childhood or adolescence than among a matched group of non-bereaved young adults who had also experienced the war during childhood or adolescence.

In the current study, we aimed at investigating levels of somatic and mental health distress as well as health care utilization among another group of bereaved civilian war survivors: widowed lone mothers. One of the studies on war-related bereavement indicated that widowed mothers might be the most vulnerable group of bereaved war survivors reporting the highest prevalence of prolonged grief among different bereaved individuals [8]. Due to the very small sample of widowed mothers in this study (n = 6), replication and extension of these results with larger samples is needed. The aim of the current study was to assess current levels of somatic, depressive, post-traumatic stress, anxiety, grief, and subjective well-being among mothers who have experienced the war as civilians, have additionally lost their husband due to war-related violence, and have been lone mothers since. Moreover, we aimed at comparing the findings from the group of widowed lone mothers with non-widowed (i.e., married) mothers. Subjective well-being, as measured in this study, is viewed as the overall evaluation of one’s quality of life [11] and may be an appropriate indicator for the assessment of adjustment and mental health in trauma survivors. A general consensus within the field of subjective well-being is that this construct consists of an affective and a cognitive dimension [12]. The affective component pertains to the feeling of balance between negative and positive affect, whereas the cognitive component comprises the satisfaction with life.

A second goal of this study was to assess utilization of primary and specialist health care services among war-related widowed and non-widowed civilian survivors of war. Assessment of utilization of these services is important given limited health care funds, especially in lower income countries which are disproportionately affected by war. Nevertheless, previous research on use of heath care services among survivors of war has mostly been carried out on veteran populations [13-16]. With regard to survivors of wars in the former Yugoslavia, preliminary data indicate that war survivors with untreated depression and posttraumatic stress disorder show a high risk of maintaining these disorders even a decade after the war [17,18]. A survey in Kosovo two years after the war revealed that use of primary health care services among war survivors was associated with posttraumatic stress disorder and number of traumatic events [19]. No study yet has investigated the role of symptoms of prolonged grief in predicting use of health care services among widowed survivors of war. The final aim of the study was to examine whether severity of prolonged grief among widowed lone mothers can significantly predict number of contacts of primary or secondary health care.

## Methods

### Study design

The current study followed a cross-sectional design and was conducted in Kosovo. After a decade of apartheid in the Kosovar Albanian population imposed by the Milosevic-led Serbian government, a full-scale war began in mid-1998 and was ended by NATO air strikes in June 1999. The study was conducted in 2009 (i.e., ten years after the war) in the region of Drenica. This central Kosovar region was chosen for this survey for feasibility reasons as the study was conducted in cooperation with a psychosocial center in Drenica. Drenica comprises of two out of 30 Kosovar municipalities (Gllogovc and Skenderaj), and 6.5% of the Kosovar population lives in this region. In the rather small country of Kosovo with two million inhabitants no significant cultural differences exist between Drenica and other regions in Kosovo. The violent conflict in Kosovo had first started in 1998 in the region of Drenica, however, it became a countrywide war within a few months affecting all regions in Kosovo. Details of the study and preliminary findings on prevalence rates of mental disorders can be found elsewhere [20]. The study was approved by the ethics committee of the University of Amsterdam.

### Participants

Exposure to at least one war-related traumatic event was an inclusion criterion for both widowed and married mothers. All potential participants were contacted directly at home without advance notification. Telephone and postal communication in Kosovo do not function effectively, especially in the countryside. Additionally, people in Kosovo have the habit of visiting each other without prior notification. For these reasons and in accordance with prior projects [21], we decided to contact potential participants at home. The study participants were informed that if they felt distressed by the interview they could seek counseling at the psychosocial center in Gllogovc. After complete description of the study to the subjects, informed consent was obtained. All participants were compensated with five Euro for their participation.

#### Group of widowed lone mothers

Complete lists of families who had lost relatives due to war-related violence in the region of Drenica were provided by communal authorities. The wives of the first 146 deceased husbands from the list were contacted, of whom 28 reported not to have children. Of the 118 potential participants, 14 widowed mothers refused to participate in the study, stating that they did not want to talk about their relatives or about the war for emotional reasons, or not having time for the interview. The rate of participation was 88.1% (104/118). Missing values resulted in the exclusion of four participants, thus 100 widowed mothers were included in the analyses. None of the widowed mothers had remarried as it is accepted custom in the Kosovar society that a widowed mother should not remarry. Thus, all 100 participants in this group were widowed lone mothers.

#### Groups of married mothers

The recruitment of married mothers took place in exactly the same localities as the recruitment of widowed mothers. A random walk approach that involved random identification of streets in each locality where bereaved participants were contacted was used to recruit non-bereaved participants as described in prior research [17]. On a particular street, every third house on the right was approached with a maximum of 15 surveys for that particular street. Of 195 households contacted for participation in the study, 74 did not meet the criterion of having children. Of the remaining 121 potential participants, 12 declined to participate in the study, indicating not having time for the survey or not wanting to talk about the war. This resulted in a participation rate of 90.1% (109/121). Missing values resulted in the exclusion of three participants. Preliminary analyses revealed that 35 mothers had reported natural death of parents or siblings after the war. In order to better be able to attribute potential differences between the groups to bereavement and its accompanying consequences, it was decided to divide married participants into two groups. The first group of married mothers consisted of 71 participants who did not report loss of family members during or after the war. This group we label non-bereaved married mothers. The second group of married mothers consisted of 35 participants who reported loss of family members (other than the husband) after the war. This group we label bereaved married mothers.

### Measures

Socio-demographic characteristics of the participants were assessed on a brief structured questionnaire. Potentially traumatic events were measured using an adjusted checklist for war-related events and is based on the first part of the Harvard Trauma Questionnaire [22]. The adjusted checklist assesses 18 potentially war-related traumatic events (such as “combat situation”, “forced evacuation under dangerous conditions”, “serious injury” or “lack of food or water”) and has been used before in the Kosovar population [8,9].

The Patient Health Questionnaire was used to measure somatic, depressive, and anxiety symptoms [23,24]. The Somatic Complaints subscale consists of 15 items, including 14 of the 15 most prevalent DSM-IV somatization disorder somatic symptoms. In the current sample, however, the majority of participants refused to answer the two items on “menstrual problems“and “sexual problems“, topics which in Kosovar society are usually considered too intimate to discuss with people other than the partner. Therefore, these two items were excluded altogether. The Depression subscale consisting of nine items was used to assess severity of depressive symptoms. Finally, the Anxiety subscale constituted of seven items was utilized to assess severity of anxiety symptoms. Studies have revealed that the PHQ has good psychometric properties [23,25,26]. In the present study, the internal consistency reliability of the Albanian version [27] of the PHQ Somatic Complaints, Depression, and Anxiety subscales were α = 0.81, α = 0.91, and α = 0.85, respectively.

Posttraumatic stress symptoms were assessed with the Posttraumatic Stress Diagnostic Scale (PDS) [28]. The PDS assesses the 17 PTSD symptoms specified in the DSM-IV. The items are scored on a 4-point Likert-type scale ranging from 0 (never) to 3 (5 times per week or more/nearly always). The PDS has shown good psychometric properties [28]. In the current study, the internal consistency of the Albanian version of the PDS [29] was α = 0.96.

Symptoms of prolonged grief among widowed lone mothers were assessed with the Prolonged Grief Disorder Interview (PGD-I) [30] that is based on the Inventory of Complicated Grief [31]. The Inventory of Complicated Grief has shown good test-retest reliability (0.80) and criterion validity [31]. The PGD-I was modified by Prigerson et al. [30] to measures the recently proposed criteria for prolonged grief [30] on a scale from 1 (“never”) to 5 (“always”). Accordingly, two items measure separation distress from the deceased one (e.g. yearning), nine items represent cognitive, emotional, and behavioral symptoms; and one item measures whether the symptoms caused “marked and persistent dysfunction in social, occupational, or other important domains.” In the current study, the internal consistency of the Albanian version of the PGD-I [8] was α = 0.76.

Subjective well-being was measured with two instruments. To measure the affective component of subjective well-being, the Positive and Negative Affect Schedule (PANAS) [32] was administered. The scale is designed to separately measure both positive and negative affect. In the current study, the short version of the PANAS was used, which consists of five items for the measurement of positive affect and five items for the measurement of negative affect [33]. Items are scored on a 5-point Likert-type scale ranging from not at all (1) to extremely (5). Furthermore, the five-item Satisfaction With Life Scale (SWLS) [34] to measure global life satisfaction was used. The 7-point scale ranges from strongly disagree (1) to strongly agree (7). The scale has proved to have highly favorable psychometric properties [34,35]. Both, the PANAS and the SWLS have been used in previous research in Kosovo [36]. The alpha coefficient of the SWLC in the current sample was α = 0.82. The alpha estimates of internal consistency of the PANAS in this sample were α = 0.86 for positive affect and α = 0.74 for negative affect.

Service use was measured using an adapted version of the Client Service Receipt Inventory [37]. This instrument has been developed for collecting retrospective information on service utilization and has a multitude of forms. In our study, participants were asked to provide details of services used during the previous three months. Services included primary health care, specialist health care, and hospitalization. Specialist health care included any sort of specialist physical health care (for e.g., gynecologist or cardiologist) and/or mental health care. Mental health care consisted of services provided by either a psychiatrist or a psychologist. Participants were first asked whether they had utilized mental health services during the previous three months. Those who reported use of health care services during the previous three months were then asked about the number of contacts and finally about the duration of contacts. Duration was recorded as following: 1 = 0-5 min; 2 = 6-15 min; 3 = 16-30 min; 4 = 31-45 min; and 5 = 46 min or longer.

The PGD-I and the Client Service Receipt Inventory were given by the interviewer. However, the interviewers were not aware of the study hypotheses. The interviews were conducted by five female psychologists who had been trained by the first author in conducting clinical interviews (for more details see [20]).

### Statistical analyses

To univariately analyze differences in socio-demographic characteristics, traumatic experiences, levels of distress and well-being, and utilization of care services between groups, x² tests, t-tests, and univariate analyses of covariance (ANCOVA) were used depending on the type of data. Linear multiple regression analyses were performed with the number of contacts of primary health use and specialist physical health use as dependent variables and demographics (age, years of education, monthly income) and number of war-related events as independent variables in the first block. Levels of depression, posttraumatic stress, and somatization were entered as independent variables in the second block. Finally, levels of prolonged grief were entered as an independent variable in the third block. Given that only three percent of widowed lone mothers and four percent of non-bereaved mothers reported utilization of mental health services during the last three months, we did not conduct regression analysis with number of contacts in mental health care. An alpha level of .05 was used for all analyses, using SPSS V18.

## Results

### Demographic and war-related characteristics

Table 1 presents the socio-demographic and war-related characteristics of the samples. As compared to non-bereaved married mothers, widowed lone mothers were significantly older (p = <.01) and reported a lower monthly income (p < .01). As compared to bereaved married mothers, widowed lone mothers were significantly older (p < .01) and had a lower level of education (p = .03). Widowed lone mothers did not differ significantly from the married groups on other socio-demographic characteristics.

**Table 1:** Socio-demographic and war-related characteristics of widowed and married mothers

	**1. Widowed lone mothers (N = 100)**	**2. Non-bereaved married mothers (N = 71)**	**3. Bereaved married mothers (N = 35)**	**Comparison between groups (ANOVA or*X***^**2**^**test)**			
				**Group 1 vs. Group 2**		**Group 1 vs. Group 3**	
				**F (df) or*X***^**2**^**(df)**	**p-value**	**F (df) or*X***^**2**^**(df)**	**p-value**
**Socio-demographic characteristics**							
Age: M (SD)	50.1 (7.9)	47.3 (6.4)	46.3 (5.8)	F(2,203) = 5.62	**0.009**	F(2,203) = 5.62	**0.006**
Years of education: M (SD)	6.1 (3.5)	6.4 (3.5)	7.5 (4.1)	F(2,201) = 2.48	0.566	F(2,201) = 2.48	**0.027**
Monthly income^1^: M (SD)	232.2 (163.3)	331.7 (233.5)	271.5 (208.4)	F(2,201) = 5.23	**0.001**	F(2,201) = 5.23	0.317
**Employment status**				*X*^2^(3) = 0.28	0.734	*X*^2^(3) = 1.50	0.681
E Employed	7 (7.0)	6 (8.5)	3 (8.6)				
U Unemployed	89 (89.0)	64 (90.1)	32 (91.4)				
R Retired	3 (3.0)	1 (1.4)	0				
Training/education	1 (1.0)	0	0				
Children in household under 18	78 (78.0)	62 (87.3)	30 (85.3)	F(2,203) = 1.40	0.059	F(2,203) = 1.40	0.075
Support from abroad	20 (20.0)	11 (15.5)	39 (22.9)	F(2,203) = 0.48	0.462	F(2,203) = 0.48	0.712
**War-related characteristics**							
Number of war traumatic events excluding murder of family M (SD)	30.7(16.14)	23.0 (18.85)	26.6 (18.6)	F(2,203) = 2.52	**0.027**	F(2,203) = 2.52	0. 324
**Displacement status during war**				*X*^2^(2) = 2.84	0.238	*X*^2^(2) = 1.86	0.396
Remained at home	3 (3.0)	5 (7.1)	1 (2.9)				
Internally displaced	65 (65.0)	49 (70.0)	27 (77.1)				
Refugee	32 (32.0)	16 (22.9)	7 (20.0)				

*Note:* Data are given as the number (percentage) of participants unless otherwise indicated; ^*1*^ Monthly income is reported in Euro.

Widowed lone mothers reported a significantly higher number of war-related traumatic events than non-bereaved married mothers (p = .03). Yet, there was no significant difference in number of war-related traumatic events between bereaved married mothers and widowed lone mothers. The three most frequently reported war-related events in all three groups were “forced evacuation under dangerous conditions” (≥ 81.7% of participants in each group), “lack of shelter” (≥ 74.7% of participants in each group), and combat situation (≥ 60.6% of participants in each group).

Pre- and post-war traumatic events were reported only by a minority of participants and there were no significant differences between the groups. Among widowed lone mothers, 16% reported more than one killing of family members during the war. Furthermore, 23% of widowed lone mothers reported death of family members after the war.

### Somatic and mental health complaints and well-being

Levels of somatic complaints, depressive, anxiety, and post-traumatic symptoms, and subjective well-being among all groups are shown in Table 2. Comparisons between groups were adjusted for age, years of education, monthly income, and number of war-related events given the significant differences among the widowed and non-widowed groups on these variables. Widowed lone mothers reported significantly higher levels on all measures of somatic and mental distress than both comparison groups. Furthermore, they reported higher levels of negative affect and lower levels of positive affect and satisfaction with life. Finally, the mean score of symptoms of prolonged grief in the group of widowed lone mothers was 50.4 (SD = 5.7).

**Table 2:** Somatic and mental health symptoms and well-being among the groups

	**1. Widowed lone mothers (N = 100)**	**2. Non-bereaved married mothers (N = 71)**	**3. Bereaved married mothers (N = 35)**	**ANCOVA**	**Comparison between groups p-value**	
	***M (SD)***	***M (SD)***	***M (SD)***	**F(2,203)**	***1*vs.*2***	***1*vs.*3***
PHQ-S	25.47 (4.86)	21.69 (5.20)	22.46 (5.04)	9.29	**<0.001**	**0.04**
PHQ-D	15.54 (7.11)	6.80 (5.81)	7.97 (6.54)	14.78	**<0.001**	**<0.001**
PHQ-A	16.32 (3.29)	12.80 (3.68)	13.14 (3.44)	11.05	**<0.001**	**<0.001**
PDS	26.96 (12.25)	8.21 (9.81)	10.18 (10.29)	22.85	**<0.001**	**<0.001**
PANAS-P	15.79 (4.71)	18.44 (4.48)	18.55 (4.32)	5.85	**<0.01**	**0.03**
PANAS-N	16.00 (3.69)	13.30 (4.87)	13.85 (3.20)	5.27	**<0.001**	**0.02**
SWLS	17.24 (6.12)	22.69 (5.62)	21.30 (6.62)	7.64	**<0.001**	**<0.01**

Note: PHQ-S: Patient Health Questionnaire-Somatization Subscale; PHQ-D: Patient Health Questionnaire-Depression Subscale; PHQ-A: Patient Health Questionnaire-Anxiety Subscale; PDS: Posttraumatic Stress Disorder Scale; PANAS-P: Positive and Negative Affect Scale-Positive Subscale; PANAS-N: Positive and Negative Affect Scale-Negative Subscale; SWLS: Satisfaction with Life Scale; Results were adujsted for age, years of education, monthly income, and number of war-related events.

### Health care utilization during the last three months

64% of widowed lone mothers, 53.3% of non-bereaved married mothers, and 51.4% of bereaved married mothers reported use of any form of health care during the previous three months. Participants in all groups reported higher use of primary health care services than other services. Only three percent of widowed lone mothers and four percent of non-bereaved married mothers (and none of bereaved married mothers) reported utilization of mental health care services during the last three months. As can be seen in Table 3, comparisons between groups did not reveal any significant difference between the groups with regard to utilization of health care services.

**Table 3:** Health care utilization during the previous three months among the groups

	**1. Widowed lone mothers (N = 100)**	**2. Non-bereaved married mothers (N = 71)**	**3. Bereaved married mothers (N = 35)**	**Comparison between groups**	
				***X***^**2**^**or F-value**	**p-value**
**Health care use**					
Utilized any health care services *N (%)*	64 (64.0)	38 (53.5)	18 (51.4)	2.68	0.26
**Primary health care**					
Utilized primary health care services *N (%)*	52 (52.0)	30 (42.3)	13 (37.1)	2.95	0.23
Number of contacts M (SD)	5.19 (4.73)	4.13 (4.00)	3.69 (2.53)	0.96	0.39
Duration M (SD)	2.84 (1.22)	2.47 (1.11)	2.31 (1.25)	1.47	0.24
**Specialist health care**					
Utilized specialist care services *N (%)*	27 (27.0)	14 (19.7)	8 (22.9)	1.23	0.54
Number of contacts M (SD)	4.33 (3.25)	3.09 (3.27)	3.13 (1.89)	0.85	0.43
Duration M (SD)	2.71 (1.37)	3.18 (1.17)	1.75 (0.89)	3.18	0.06
Utilized physical health care services *N (%)*	24 (24.0)	11 (15.5)	8 (22.9)	1.92	0.38
Utilized mental health care services *N (%)*	3 (3.0)	3 (4.2)	0 (0)	1.49	0.48
**Hospital**	4 (4.0)	0 (0)	1 (2.9)	2.83	0.24

Finally, two hierarchical linear regression analyses were performed among widowed lone mothers to examine the role of symptoms of prolonged grief in predicting use of 1) primary care or 2) specialist physical care services. At step one, age, years of education, monthly income, and number of war-related events were entered. At step two, scores of depression, posttraumatic stress, and somatization were entered. At step three, scores of prolonged grief were entered. When number of contacts of primary care use was entered as the dependent variable, demographics and number of war-related variables accounted for significant variance, levels of depression, posttraumatic stress, and somatization accounted for additional variance, and levels of prolonged grief did not account for additional variance (see Table 4 for details). With regard to number of contacts of specialist physical care use as the dependent variable, neither demographics and number of war-related variables in step one, nor levels of depression, posttraumatic stress, and somatization accounted for significant variance. When levels of prolonged grief were entered in the third step, only prolonged grief accounted for significant variance in number of specialist care use (s. Table 4).

**Table 4:** Hierarchical regression for variables predicting number of contacts in primary and specialist health care among widowed mothers

***Outcome: number of contacts in primary health care***							
**Step**		**B**	**SE**_**b**_	**β**	**T**	**ΔR**^**2**^	**ΔF**
1	Age	.01	.06	.02	.20	.11	2.96*
	Years of education	.19	.13	.16	1.49		
	Monthly income	.004	.003	.14	1.41		
	Number of war-related events	.60	.03	.22	2.20*		
2	PHQ-D	.15	.07	.25	2.12*	.19	8.35***
	PDS	.03	.04	.08	.83		
	PHQ-S	.47	.10	.54	4.78***		
3	PGD-I	.11	.08	.15	1.37	.01	1.88
*Outcome: number of contacts in specialist physical health care*							
Step		B	SE_b_	β	T	ΔR^2^	ΔF
1	Age	.04	.04	.12	1.06	.08	1.96
	Years of education	.13	.08	.18	1.69		
	Monthly income	.001	.002	.09	.88		
	Number of war-related events	.001	.02	.01	.08		
2	PHQ-D	.01	.05	.03	.19	.03	0.92
	PDS	.01	.02	.04	.38		
	PHQ-S	.07	.06	.14	1.11		
3	PGD-I	.14	.05	.33	2.80**	.07	7.83**

Note: PHQ-D: Patient Health Questionnaire-Depression Subscale; PDS: Posttraumatic Stress Disorder Scale; PHQ-S: Patient Health Questionnaire-Somatization Subscale; PGD-I: Prolonged Grief Disorder Interview. * *p* < .05. ** *p* < .01; *** *p* < .001.

## Discussion

A decade after experiencing war-related events, widowed lone mothers reported significantly higher levels of somatic complaints, depression, anxiety, and post-traumatic stress than non-bereaved married mothers and bereaved married mothers even after adjusting for socio-demographic and war-related variables. Accordingly, widowed lone mothers reported significantly lower scores of subjective well-being than the two groups of married mothers. The groups did not differ with regard to utilization of health care services during the previous three months.

To our knowledge, this is the first study to make a direct comparison of levels of somatic and mental health distress and subjective well-being between war-related widowed lone mothers and married mothers, all of whom had experienced the war first hand. The results are strengthened by the fact that the comparison groups were recruited in exactly the same localities as widowed lone mothers. Yet, the study has a few limitations. The recruitment of participants in only one region limits the generalization of the findings to other populations with war-related bereavement. The cross-sectional design does not allow any conclusions on causal associations between the measured variables. Further, current findings on the differences and associations of the measured variables might still be subject to unobserved confounding war-related and postwar-related factors.

Of particular interest is the finding that widowed lone mothers reported higher levels of somatic and mental health complaints and lower levels of psychological well-being not only as compared to non-bereaved married mothers but also to bereaved married mothers. The finding that widowed lone mothers reported significantly higher levels of depression, somatization, anxiety, and posttraumatic stress than bereaved married mothers even after adjusting for relevant variables suggests that war-related killing of the husband and its accompanying consequences (first and foremost lone motherhood) might constitute a significant risk for psychopathology above and beyond other war-related traumatic events and bereavement following natural loss. This finding is strengthened by the fact that bereaved married mothers had experienced loss of first-degree relatives after the war. Thus, more time had passed since the killing of the husband of widowed lone mothers than since the loss of family members among bereaved married mothers, yet widowed lone mothers still reported higher rates of psychopathology. These findings are also in line with findings related to prevalence rates of major depressive episode, post-traumatic stress disorder, generalized anxiety disorder, and suicide risk resulting from this study that have been reported elsewhere [20]. Widowed lone mothers reported significantly higher rates of posttraumatic stress disorder (82%), major depressive episode (71%), generalized anxiety disorder (48%) and suicide risk (45%) than non-bereaved (29.6%, 18.3%, 9.9% and 16.9%, respectively) and bereaved married mothers (25.7%, 25.7%, 25.7% and 22.9%, respectively).

Severity of psychopathology among widowed lone mothers is high in comparison to existing literature on survivors of war. For instance, in a recent study with war-related bereaved civilian survivors of war from Kosovo that included only ten percent widowed female war survivors and that used the same instrument to measure posttraumatic stress symptoms as in the current study (PDS), a mean score of posttraumatic stress symptoms of 18.08 was reported [8] as compared to a mean score of 29.96 in the current study. The levels of somatic complaints among widowed lone mothers in this study are also very high in comparison to a recent study conducted in Kosovo with young adult war survivors with and without loss of father due to war-related violence (M = 25.74 vs. 18.4 and 17.8, respectively) [9]. Severity of prolonged grief was also higher in the current study (M = 50.4) than in other studies with survivors of war that have used the same instrument. In the above mentioned study with Kosovar young adult war survivors with war-related loss of father [9], a mean of 41.7 was reported. In another study [8], a mean score of 28.7 was reported among war-related bereaved civilian survivors of war from Kosovo that had reported war-related loss of a first-degree relative. Similarly, the prevalence rate of prolonged grief disorder (69%, see [20]) was higher than in the above mentioned studies conducted in Kosovo as well as in a recent study in survivors of the 1994 Rwandan genocide with loss of a parent or husband before, during, or after 1994 that reported a prevalence rate of prolonged grief disorder of 8% [10].

The higher levels of severity of mental health and somatic distress among widowed lone mothers who remain lone mothers indicate that this population is particularly at risk for increased psychopathology. These findings are in line with results from bereavement research indicating that bereavement is associated with elevated general health distress as compared to non-bereaved groups [3]. More importantly, however, the findings are supported by pilot studies among survivors of war indicating that bereaved survivors report higher prevalence rates of mental disorders than non-bereaved participants [9,38]. Likely wise, our findings are consistent with pilot results from a recent study conducted in Kosovo indicating that war-related widows report higher psychiatric sequelae than other bereaved participants [8]. Finally, the finding in our study that severity of prolonged grief was the only significant predictor of specialist physical health care further strengthens the relevance of research into prolonged grief, including war-related prolonged grief.

Several factors such as emotional, functional, and economic difficulties are assumed to play a role in elevated levels of psychopathology. Widowed lone mothers must cope with their own experiences of war, killing of the husband during the war, being a lone mother, and raising children on their own. Loss of the husband due to war-related violence might contribute to psychopathology in different ways, such as the loss of the husband with whom the widow had a strong emotional relationship as a partner, the traumatic circumstances under which the loss of the husband took place, or the loss of the father of the children of the widow. In post-war Kosovar society, loss of the husband meant also loss of the main breadwinner and was accordingly associated to economic difficulties. This is also reflected by the reported unemployment rate of 89% among widowed lone mothers. Although the survey did not differentiate between those who were seeking employment and were unable to find it and those who did not want to work, this extremely high unemployment rate still revealed that 89% of widowed lone mothers did not have a wage-earning job. While war-related widowed women in Kosovo receive social welfare benefits and often also immaterial help from relatives, the total monthly income of the bereaved families in this study was significantly lower than that of married participants. The accepted norm in Kosovar society that a widowed mother should not remarry, resulting into forced lone motherhood, is likely to negatively affect coping mechanisms following exposure to war-related events and the killing of the husband and to hamper attempts at mastering socioeconomic circumstances. Thus, lone motherhood is likely to constitute a significant factor in the elevated rates of psychopathology. In fact, research in Western countries has indicated that lone motherhood is associated with poorer psychological health and higher risk of mortality as compared to partnered motherhood [39-41]. Further factors that might influence mental health among this population are on-going societal instability, political uncertainty, and unsatisfactory health and social care. This notion is in line with findings from studies conducted with survivors of war living in the countries of former conflict [17] as well as among refugees revealing that post-migration living stressors contribute to psychopathology in addition to war- or torture related events [42-45]. Yet, the interaction between exposure to war-related events, post-war practical difficulties, and mental health status among war survivors living in the areas of former conflict is still only poorly understood and needs further examination. Additionally, the impact of the traumatic circumstances under which the husband was killed on psychopathology needs further empirical investigation.

The fact that widowed lone mothers reported higher number of war-related traumatic events than non-bereaved married mothers needs further explanation. Checklists of traumatic events such as the one used in this study pose a limitation with regard to comparing number of traumatic events among groups exposed to several traumatic events. The very nature of single traumatic events cannot be captured with the existing checklists of traumatic events. This indicates that even after controlling for number of traumatic events in the current study, health distress differences between widowed lone mothers and the comparison groups might exist not only due to the consequences of losing the husband and being a lone mother but also due to the experience of other war-related events. Furthermore, 16% of widowed lone mothers reported war-related loss of first-degree relatives other than the husband as compared to none in the control groups. Although we recruited both bereaved and non-bereaved participants in the same localities, killing of the husband seemed to have increased the likelihood of being exposed to more traumatic events and also losing other family members as compared to participants whose husbands were not killed during war. Our findings should be seen as preliminary and need replication.

Civilian war survivors experience a wide array of potentially traumatic events that may have diverse consequences in terms of morbid psychiatric outcomes [46]. Therefore, future studies need to further investigate the associated psychological consequences of single war events and mental well-being. For example, research has shown that torture and rape are also associated with more general health distress as compared to other war experiences [47,48]. War-related death of the husband is another case in point worth of further study.

More than 50% of participants in all groups reported any use of health care services during the last three months. However, the majority of participants reported primary health care utilization only and barely a tiny proportion of participants reported utilization of mental health services during the last three months. Participants were not asked about reasons for seeking and especially not seeking health care services. Furthermore, they were also only asked about utilization of health care during the last three months. However, given the high levels of mental and physical distress especially in the widowed group, it is likely that participants had little or no hope in finding any useful mental health care. This corresponds with reports that in Kosovo primary health care was given special focus following the war in 1998/1999 and that specific interventions for treating mental disorders have only rarely been available [49]. Further possible reasons for low rates of mental health care use might be related to stigmatization of seeking mental health care or fear of recalling painful experiences.

Recent research with survivors of war seeking mental health treatment in the Balkans [50], including Kosovo [51], has shown that utilization of mental health services in these countries is not associated with improved levels of mental health. These findings might further explain why a very small proportion of our participants reported use of mental health services despite elevated levels of mental health and somatic distress. Given the fact that utilization of mental health care services is an interplay of availability of resources, need for those resources as well as individual and social characteristics [52], an advancement of mental health services for survivors of war as well as a better understanding of possible barriers for seeking mental health needs seem obligatory. Recent research in Kosovo has also found that posttraumatic stress disorder and major depression were not significantly associated with medical visits or hospitalization in the past 12 months [52].

## Conclusion

The high levels of psychopathology among war-related widowed lone mothers and the lack of appropriate mental health services call for long-term policies to meet their special mental health needs. Preventing violations to human rights is a humanitarian challenge for the international community. Yet, with many ongoing violent conflicts leading to a steady rise of war-related bereavement the international community also has an obligation to investigate and attenuate the psychological consequences of human rights violations. This appears especially important in post-war societies which themselves lack the ability to offer sufficient mental care to those in need.

## Competing interests

The authors declare that they have no competing interests.

## Authors’ contributions

NM carried out the study, participated in its design and its coordination, performed the statistical analyses and drafted the manuscript. PMGE participated in the design of the study and contributed to the interpretation of findings and writing of the manuscript. All authors read and approved the final manuscript.

## Pre-publication history

The pre-publication history for this paper can be accessed here:

http://www.biomedcentral.com/1471-244X/12/39/prepub
